# Hodgkin Lymphoma Monozygotic Triplets Reveal Divergences in DNA Methylation Signatures

**DOI:** 10.3389/fonc.2020.598872

**Published:** 2020-12-09

**Authors:** Chuanyou Xia, Thale Kristin Olsen, A. Ali Zirakzadeh, Radwa Almamoun, Louise K. Sjöholm, Jenny Dahlström, Jan Sjöberg, Hans-Erik Claesson, John Inge Johnsen, Ola Winqvist, Dawei Xu, Tomas J. Ekström, Magnus Björkholm, Klas Strååt

**Affiliations:** ^1^ Department of Medicine, Division of Hematology, BioClinicum and Centre for Molecular Medicine, Karolinska University Hospital Solna and Karolinska Institutet, Stockholm, Sweden; ^2^ Childhood Cancer Research Unit, Department of Women’s and Children’s Health, Karolinska Institutet, Stockholm, Sweden; ^3^ Unit of Translational Immunology, Department of Medicine, Karolinska Institutet, Stockholm, Sweden; ^4^ Department of Clinical Neuroscience, Karolinska Institutet, Center for Molecular Medicine, Stockholm, Sweden

**Keywords:** Hodgkin lymphoma, monozygotic triplets, DNA methylation, marginal zone-like B-cells, CD34+ cells, naïve B cells, switched memory B-cells

## Abstract

We studied DNA methylation profiles in four different cell populations from a unique constellation of monozygotic triplets in whom two had developed Hodgkin Lymphoma (HL). We detected shared differences in DNA methylation signatures when comparing the two HL-affected triplets with the non-affected triplet. The differences were observed in naïve B-cells and marginal zone-like B-cells. DNA methylation differences were also detected when comparing each of the HL-affected triplets against each other. Even though we cannot determine whether treatment and/or disease triggered the observed differences, we believe our data are important on behalf of forthcoming studies, and that it might provide important clues for a better understanding of HL pathogenesis.

## Introduction

We previously reported Hodgkin lymphoma (HL) development in two monozygotic triplets where all three were homozygotic through a constitutional deletion in the first intron of the megakaryoblastic leukemia 1 gene *(MKL1)* (1). Two of the HL affected triplets were diagnosed with HL at the age of 40 and 63, respectively. Clinical details of the triplets are presented in reference (1) and in supplementary material. The significance of the MKL1 gene deletion in the same set of triplets has recently been studied further by Record et al (2). In this paper, we showed that lymphoblastoid cell lines derived from the HL-free triplet had higher MKL1 mRNA and protein levels when compared to the HL affected triplets, while cells derived from the HL treated triplets resembled the healthy controls used in the study. Additionally, we could show that cells from the HL-free sibling had increased cell proliferation, a larger number of hyperploid cells, and further, that cells derived from this triplet were able to form large tumors *in vivo*. This indicates that the HL-free sibling has pre-malignant cells emerging while the HL-affected triplets do not, possibly due to previous treatment. Together, these data suggest that dysregulated MKL1 activity may participate in B cell transformation and HL pathogenesis (2). After our initial report of the HL triplet cases (1), we have not seen any other publications addressing the role of *MKL1* deletions in HL pathogenesis.

It is well recognized that first-degree relatives of HL-patients carry an increased risk of developing HL (3) and that the risk increases in monozygotic twins (4). HL tumors have unique features since the tumor tissue only consists of a minute number of the actual cancer cells which are called Hodgkin/Reed-Sternberg (HRS) cells. These cancerous cells are believed to originate from a germinal or post germinal center B-cell. The rest of the tumor microenvironment mainly harbors inflammatory cells of the immune system, such as lymphocytes, eosinophils, neutrophils, histocytes, plasma cells, and dendritic cells, reviewed in (5, 6).

DNA methylation is thought to play an important role in carcinogenesis. Hypermethylation in CpG islands of promoters is assumed to facilitate the silencing of gene transcription. Indeed, hypermethylation of B-cell specific genes such as *CD79B, BOB1, SYK, PU.1, CD19, and CD20* is implicated in HL (7).

Previous studies carried out on these triplets used peripheral blood mononuclear cells (PBMCs) collected from whole blood with no specific selection of cells (1), or isolated CD19+ B-cells transformed into lymphoblastoid cells (2).

## Methods

PBMCs were isolated using density centrifugation with Ficoll-Paque (GE Healthcare). The number of cells was counted before they were stored in AIM V medium (Gibco) in +4°C overnight. CD34+ cells were sorted out by positive selection using the CD34+ isolation kit (Miltenyi). After CD34+ cell separation, B-cells were sorted from the PBMCs using the CD20^+^ positive selection isolation kit (Miltenyi), naïve B-cells, marginal zone-like B cells, and switched memory B-cells were sorted using FACS Aria (BD). Genomic DNA (gDNA) was isolated from CD34+ cells and naïve, marginal zone-like and switched memory B-cells using the DNeasy Blood & Tissue Kit (Qiagen) in accordance with the manufacturer’s instructions.

Subsequently, genome-wide DNA methylation was analyzed using the Illumina EPIC array with 850.000 CpG site-specific probes. Raw.idat files were analyzed using the minfi R package (version 1.28.4) (8). Beta and M values were extracted and preprocessed using the preprocessFunnorm command. Loci with known SNPs and/or detection p values > 0.01 were filtered from subsequent analyses. For each of the four cell types, pairwise comparisons between individuals (A vs. B, A vs. C, B vs. C) were made using the DMRforPairs R package (version 1.18.0) (9). Results were then cross-checked to identify all regions that were differentially methylated between the non-affected triplet sample C and each of the HL-affected samples A and B. In the analysis, default settings were used as described in the DMRforPairs vignette. Microarray data are available at GEO under accession number GSE142202.

The study was approved by the local ethics committee, Dnr: 2008/1764-31, and informed consent was given before samples were collected. Methods and bioinformatic analysis in more detail can be found in the supplementary material.

## Results

**Figure 1:**
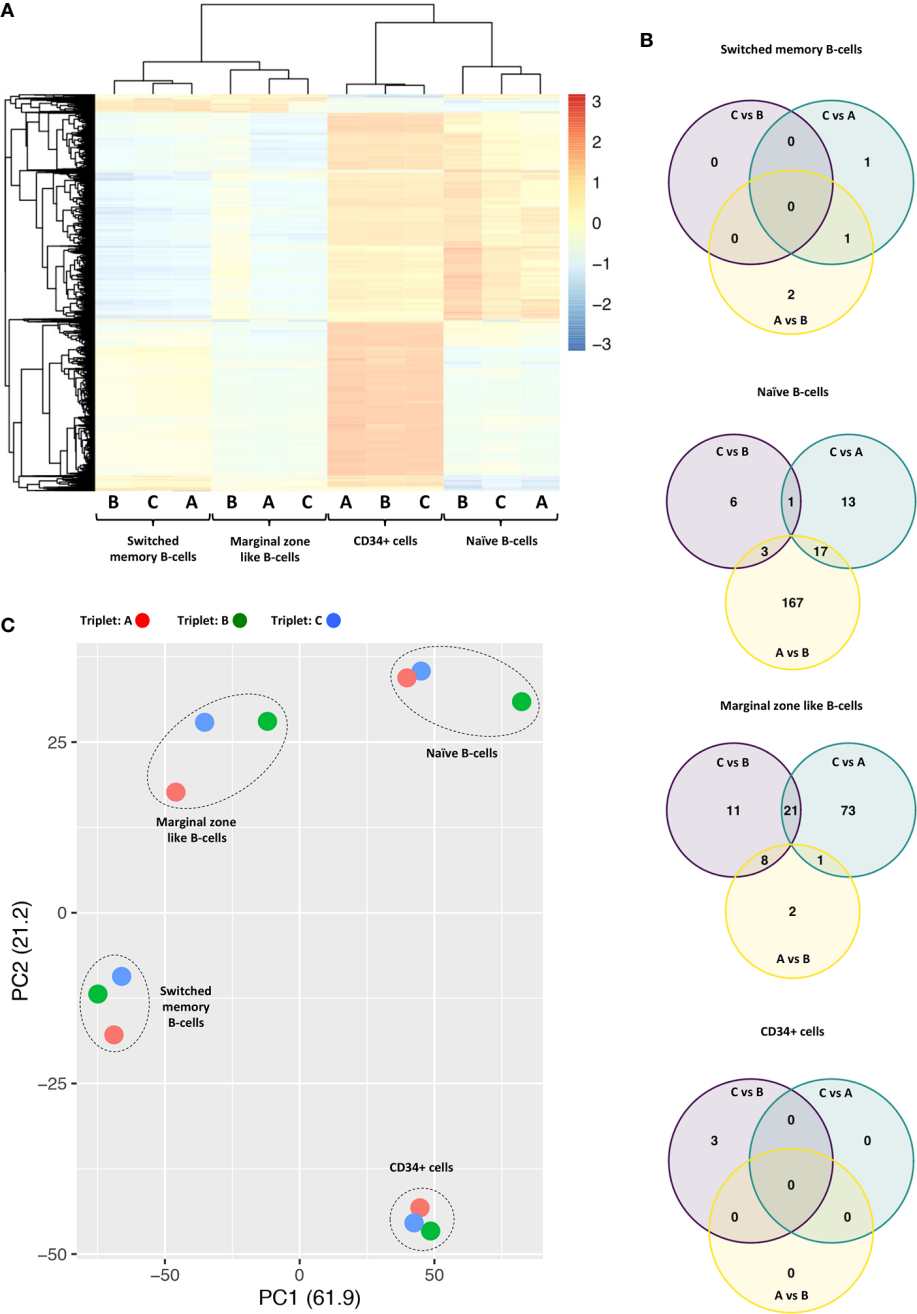
**(A)** Unsupervised hierarchical clustering of methylation levels from the 10,000 most variable probes across all samples derived from HL-affected triplets A, B, and non-affected triplet C Probe variability is quantified by standard deviation. M values are scaled by row (z score); red color indicates M values above mean; blue color indicates M values below mean. Samples from the four cell types primarily cluster by cell type. **(B)** Venn diagrams showing overlaps of differentially methylated regions when each triplet is compared to the others in pairwise comparisons. Of particular interest is the overlap between the “C vs. B” and the “C vs. A” sets (n =1 for naïve B cells; n = 21 for marginal zone-like B cells). **(C)** Principal component analysis (PCA) of all samples. PC1 and PC2 explain 61.9% and 21.2% of variability, respectively. Each triplet is labeled by color and each cell type is indicated in the plot.

Unsupervised hierarchical clustering demonstrated that the four cell types studied were distinct from each other in terms of methylation levels (**Figure 1A**). This cell type distinction was also reflected in the principal component analysis (PCA) presented in (**Figure 1C**). When comparing the methylation profile of each HL-triplet against the non-HL-triplet, shared differences in DNA methylation were found in naïve B-cells and marginal zone-like B-cells (**Figure 1B**). In contrast, we found no shared changes in DNA methylation in switched memory B-cells or CD34+ cells. A list of genes located in close proximity to these differentially methylated CpG loci is presented in **Table 1**.

**Table 1:** List of genes with shared differences between the two HL-affected triplets as compared to the non-affected sibling.

Cell type	Chr	Start	End	Length (bp)	Gene/Symbol of overlapping transcripts (within 10,000 bp)
Naïve B cells	18	64271341	64271643	303	*CDH19*
Marginal zone-like	1	200860363	200860537	175	*C1orf106*
	1	225117389	225117464	76	*DNAH14*
	2	46465	46752	288	*FAM110C*
	3	3841043	3841252	210	*LRRN1, SUMF1*
	3	129693370	129693791	422	*TRH, RP11-93K22.6*
	3	138067053	138067623	571	*MRAS*
	4	11430353	11430903	551	*HS3ST1*
	4	55991818	55991943	126	*KDR*
	4	77227489	77227631	143	*FAM47E-STBD1, CCDC158*
	4	90758207	90758537	331	*RP11-67M1.1, SNCA*
	5	9546271	9546404	134	*SNHG18, SNORD123, SEMA5A*
	5	128300967	128301185	219	*SLC27A6*
	8	26371428	26371573	146	*BNIP3L, DPYSL2, PNMA2*
	15	35047023	35047411	389	*RP11-814P5.1, GJD2*
	15	57668539	57668554	16	*CGNL1*
	20	13200931	13200992	62	*ISM1*
	22	20792143	20792535	393	*SCARF2, KLHL22*
	X	101905837	101906537	701	*RP4-769N13.6, GPRASP1, RP4-769N13.7*

In the naïve B-cell subpopulation, we found one region on chromosome 18 that differed in DNA methylation. Here, samples from both HL-affected triplets displayed hypermethylation when compared with the non-affected triplet. In marginal zone-like B-cells, we found 21 genomic regions on 10 different chromosomes which were all hypermethylated in both of the HL-affected triplets as compared to the non-affected triplet. In CD34+ and switched memory B cells, we found no common regions that differed in methylation signature (**Figure 1B**). The identified probe regions and corresponding genes in the studied cell populations are listed in detail in **Table 1** and **Supplementary Material**.

## Discussion

Several genes related to HL, cancer or hematopoiesis are located in or near the differentially methylated regions in marginal zone-like B-cells. For example, *ISM1* has been detected in lymphocytes, bone marrow, and embryonic blood islands and found to contribute to the hematopoiesis in zebrafish (10). *KLHL22* is likely involved in tumorigenesis through the activation of the amino-acid-dependent mTOR pathway (11). *SNHG18* was identified to play as a tumor suppressor gene in hepatocellular carcinoma (12). Recent studies have shown that exogenous expression of *SNCA* through the transcription factor ΔNp63α could induce the migration of breast cancer cells (13). Down-regulation of *SNCA* is also associated with ovarian cancer drug resistance (14). *MRAS* is known to play a key role in tumor growth (15), and it contributes to the activation of several important signalling cascades such as the MAPK and ERK pathways. *KDR* is known to regulate angiogenesis, vascular development, vascular permeability, and embryonic hematopoiesis. In a cohort study (16), including 17 HL-prone families, a predisposing mutation (p.A 1065T) in the *KDR* gene was identified and suggested to enhance tumor proliferation.

We could also detect differences in DNA methylation when comparing the HL-affected triplets against each other. Similarly, we observed shared differences between the HL-affected triplet and the non-affected triplet (**Figure 1B**). Such differences might occur due to inter-individual variation, environmental factors, or stochastic events. Nevertheless, we believe that the results obtained here are of potential value in forthcoming studies related to DNA methylation and pathogenesis of HL.

The identified DNA hypermethylation could possibly govern a lower expression of affected genes. Recent studies suggest that inherited leukemia-associated somatic mutations might have a low impact on clonal hematopoiesis (CH) (17, 18), suggesting that DNA methylation has a larger impact on CH than anticipated. Thus, it would be of interest to further investigate DNA methylation in more detail in patients with hematological diseases. Studies of DNA methylation in patients with various hematological disorders will probably increase our understanding of their pathogenesis. Our and others’ results emphasize the importance of understanding the role of genome-wide DNA methylation in malignancies.

We are well aware that the study of this unique triplet constellation has its limitations regarding statistical strength. Even though the two HL patients A and B ended terminated their chemotherapy treatment 33 and 8 years, respectively, before samples were collected, we cannot fully rule out that chemotherapy has contributed to the observed differences in DNA methylation. This would be important to know since it would demonstrate that established methylation alterations in the adult can be sustained over very long periods.

The two HL triplets described in this study were 40 and 63 years of age at the time of diagnosis, and both cases were EBV positive (1). The familiar risk of HL in siblings as well as monozygotic twins is primarily associated with early-onset disease (3, 4). Although heritability of EBV-positive HL in older patients is not clearly established (19), we cannot rule out heritable factors contributing to disease in these cases.

There are studies indicating that chemotherapy has an effect on DNA-methylation. For example, a study on short-term (4 months) effects of chemotherapeutic treatment showed that leukocyte DNA-methylation was altered by chemotherapy in breast cancer patients (20). In this study, CpG islands in or near *VMP1, CORO1B, SDK1*, and *SUMF2* were found to be significantly altered after chemotherapy. None of these genes were differentially methylated in our study. However, the *SUMF2* paralog *SUMF1* was hypermethylated in the marginal zone-like cells of HL affected triplets (**Table 1**). SUMF2 is known to inhibit the sulphatase-enhancing activity of SUMF1 (21). It is possible that chemotherapy might influence expression of *SUMF2/SUMF1* and thus the regulation of sulphatase activity in cells. It will be of great interest to see if similar findings may be observed by others in twins or triplets discordant for the diagnosis of HL.

In summary, we report a unique constellation of monozygotic triplets where DNA methylation levels in marginal zone-like B-cells and naïve B-cells were observed to be different in two HL-affected triplets as compared to the non-affected triplet. These differences in DNA methylation levels may contribute to HL-pathogenesis.

## Data Availability Statement

The original data presented in this study is publicly available at the Gene Expression Omnibus (GEO), accession number GSE142202.

## Ethics Statement

The studies involving human participants were reviewed and approved by Etikprövningsnämnden Stockholm. The patients/participants provided their written informed consent to participate in this study.

## Author Contributions

CX interpreted data and wrote the manuscript. TO performed and interpreted bioinformatic analysis and took part in writing the manuscript. AZ and OW performed FACS sorting to isolate the different cell types analyzed in the study. RA and LS prepared DNA samples for analysis by Illumina EPIC. JS, H-EC, JJ, DX, and TE took part in interpreting data and reviewing the manuscript. MB, DX, and TE took part in the design of the study, interpreted data, and reviewed the manuscript. KS took part in the design of the study, interpreted obtained results as well as writing the manuscript and preparing figures and tables. All authors contributed to the article and approved the submitted version.

## Funding

This study was supported by grants from Swedish Cancer Society, Cancer Society in Stockholm, Stockholm County Council and the Swedish Research Council. PhD student CX was partially supported by a PhD scholarship from Chinese Scholarship Council. The authors declare that this study received funding from Takeda Pharma AB. The funder was not involved in the study design, collection, analysis, interpretation of data, the writing of this article or the decision to submit it for publication.​​​​​

## Conflict of Interest

The authors declare that the research was conducted in the absence of any commercial or financial relationships that could be construed as a potential conflict of interest.
