# Supplementary material for: Hodgkin Lymphoma Monozygotic Triplets Reveal Divergences in DNA Methylation Signatures
Source: Front Oncol. 2020 Dec 9;10:598872. doi: 10.3389/fonc.2020.598872 (PMC7756121; doi:10.3389/fonc.2020.598872)
Supplement: Supplementary file 1 [file DataSheet_1.pdf]

Supplementary data

Materials and methods

Isolation of cells

PBMCs were isolated from the blood of male triplets of which two is diagnosed with Hodgkin Lymphoma but were now symptom-free, using density centrifugation with Ficoll Paque (GE Healthcare). The number of cells was counted before they were stored in AIM V medium (Gibco) in +4°C overnight.

Triplet A (HL): 45 x 10^{6}

Triplet B (HL): 29 x 10^{6}

Triplet C (Non-affected): 30 x 10^{6}

The next day, CD34+ cells were sorted out by positive selection using the CD34+ isolation kit (Miltenyi). The number of isolated CD34+ cells were counted:

Triplet A (HL): 2.6 x 10^{5}

Triplet B (HL): 2.5 x 10^{5}

Triplet C (Non-affected): 5.7 x 10^{5}

After CD34+ cell separation, B-cells were sorted from the PBMCs using $CD20^{+}$ positive selection isolation kit (Miltenyi). The number of $CD20^{+}$ cells was counted:

Triplet A (HL): 2.2 x 10^{6}

Triplet B (HL): 3.2 x 10^{6}

Triplet C (Non-affected): 2.6 x 10^{6}

$CD20^+$ cells were then labeled with CD19 (APC-Cy7), IgD (FITC) and CD27 (PE) antibodies (BD). Naïve (IgD^+ $CD27^-$), marginal zone-like (IgD^+ $CD27^+$) and switched memory (IgD^- $CD27^+$) -B-cells were sorted using FACS Aria (BD). The number of sorted cells is given in the table below:

	Naïve B-Cells	Switched Memory B-Cells	Marginal Zone-like B-Cells
Triplet A (HL)	3.5×10^5	6.0×10^5	2.0×10^5
Triplet B (HL)	7.5×10^5	5.0×10^5	1.0×10^5
Triplet C (Non-affected)	1.5×10^5	7.8×10^5	2.0×10^5

The purity of the B-cell subtypes was investigated and given in the table below:

	% Naïve B-Cells	% Switched Memory B-Cells	% Marginal Zone-like B-Cells
Triplet A (HL)	98.7	95.2	92.1
Triplet B (HL)	99.2	96.4	83.0
Triplet C (Non-affected)	98.9	96.7	92.7

A graph of cell-specific purity is presented in Supplementary Figure 1.

All cells were pelleted and stored in -80° C.

DNA isolation and bisulfite treatment procedures.

Genomic DNA (gDNA) was isolated from CD34+ cells and Naïve, marginal zone-like and switched memory B-cells, by using the DNeasy Blood & Tissue Kit (Qiagen), in accordance

with the manufacturer's instructions. gDNA concentrations were determined spectrophotometrically. Bisulfite conversion was performed using the EZ DNA MethylationTM Kit D5004 (Zymo Research) with approximately 250 ng of DNA per sample. The bisulfite converted DNA was eluted in 15µl according to the manufacturer´s protocol, evaporated to a volume of <4µl, and used for methylation analysis using the Illumina Methylation EPIC array.

Bioinformatic analysis.

Idat files from Illumina EPIC human methylation array were analyzed using the *minfi* R package (version 1.28.4). Beta and M values were extracted, processed and normalized using the *preprocessFunnorm* command with settings nPCS = 2, sex = male, bgCorr = TRUE, and dyeCorr = TRUE. Loci with known SNPs and/or detection p values > 0.01 were filtered from subsequent analyses. For each of the four cell types, three pairwise comparisons between samples were made Triplet A (HL) vs non-affected triplet C; Triplet B (HL) vs non-affected Triplet C, and Triplet A (HL) vs Triplet B (HL). Comparisons were made using the *DMRforPairs* R package (version 1.18.0) with default settings as described in the vignette. Regions with significant differences in methylation between triplets were then cross-checked to identify all regions that were differentially methylated between the non-affected triplet sample C and each of the HL-affected samples A and B. Methylation data was uploaded to GEO, accession no is GSE142202.

Supplementary table 1 Clinical details about the studied triplets.

**Triplet:**	**A (HL)**	**B (HL)**	**C (Non-affected)**
Age at diagnosis	40	63	/
EBV status*	Positive	Positive	/
HL subtype	Mixed cellularity	Nodular sclerosis	/
Treatment	Mustargen, oncovin, procarbazine, prednisone/adriamycin, bleomycin, vinblastine, and dacarbazine chemotherapy (MOPP/ABVD)	Adriamycin, bleomycin, vinblastine and dacarbazine chemotherapy. (ABVD)	/
Current status	Complete remission	Deceased due to cardiac insufficiency (free from HL at time of death)	Non affected

* Triplet A and B confirmed EBV DNA positive in tumor cells.

Supplementary figure 1

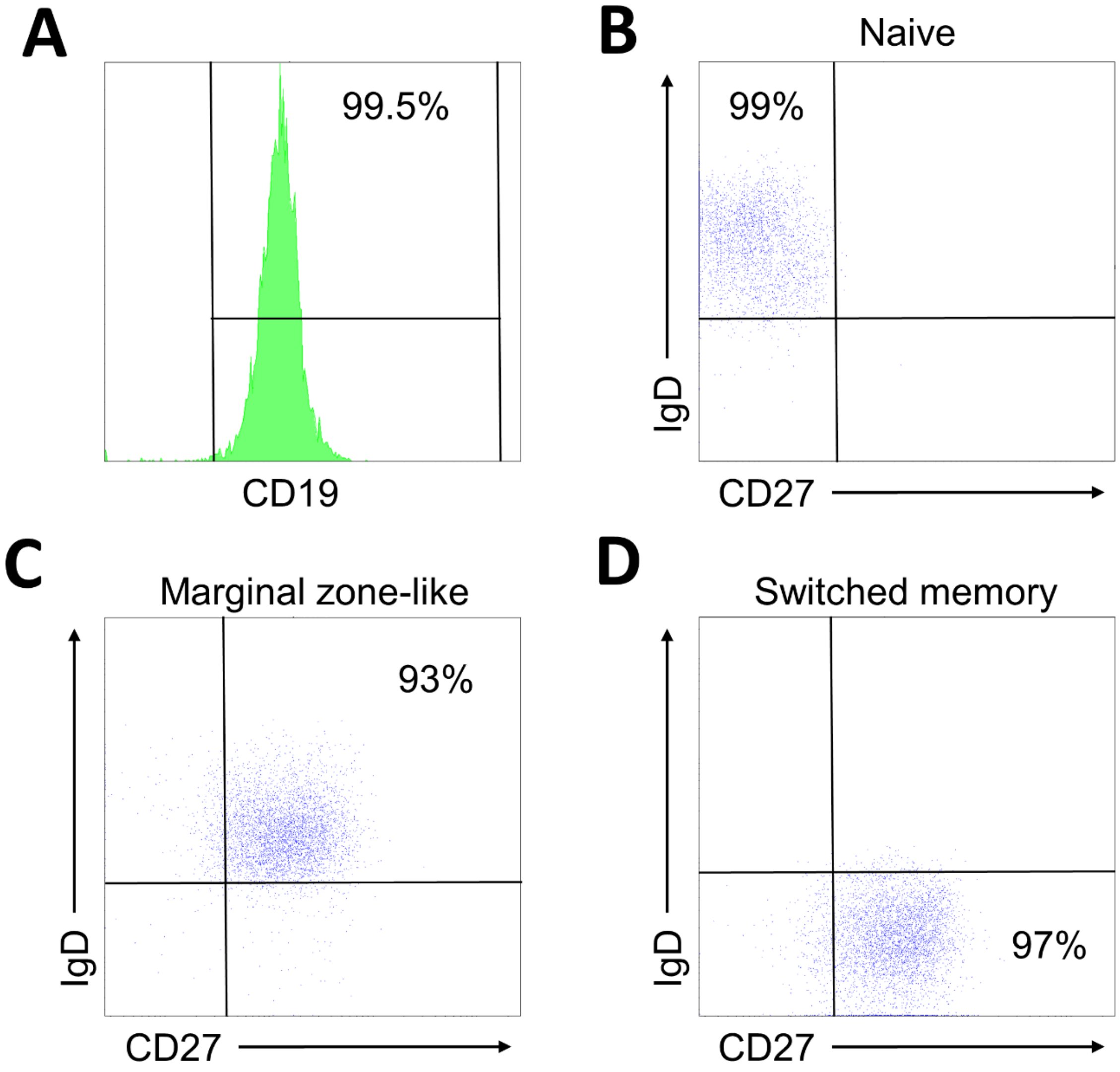

Figure legends

Supplementary figure 1

FACS plot over the purity of the different B-cell subtypes after CD34+ selection.
